# International Medical Annual for 1894

**Published:** 1894-01

**Authors:** 


### International Medical Annual for 1894.

E. B. Treat, Publisher, New York, has in press for early publication the 1894 *International Medical Annual*, being the twelfth yearly issue of this eminently useful work. Since the first issue of this one volume reference work, each year has witnessed marked improvements, and the prospectus of the forthcoming volume gives promise that it will surpass any of its predecessors. It will be the conjoint authorship of forty-one distinguished specialists, selected from the most eminent physicians and surgeons of America, England, and the Continent. It will contain complete reports of the progress of medical science in all parts of the world, together with a large number of original articles and reviews on subjects with which the authors' names are especially associated. In short, the design of the book is, while not neglecting the specialist, to bring the general practitioner into direct communication with those who are advancing the science of medicine, so he may be furnished with all that is worthy of preservation, as reliable aids in his daily work. Illustrations in black and colors will be consistently used wherever helpful in elucidating the text. Altogether it makes a most useful if not absolutely indispensable investment for the medical practitioner. While the book will be so much improved over previous issues, the price will remain the same as heretofore, $2.75.

